# Neutrophil extracellular traps activate lung fibroblast to induce polymyositis‐related interstitial lung diseases via TLR9‐miR‐7‐Smad2 pathway

**DOI:** 10.1111/jcmm.14858

**Published:** 2019-12-10

**Authors:** Sigong Zhang, Xueqin Jia, Qiuyue Zhang, Li Zhang, Jing Yang, Caihong Hu, Junnian Shi, Xiao Jiang, Jinyue Lu, Haili Shen

**Affiliations:** ^1^ Department of Rheumatology Lanzhou University Second Hospital Lanzhou China; ^2^ The Second Clinical Medical College Lanzhou University Lanzhou China; ^3^ Department of Endocrinology Lanzhou University Second Hospital Lanzhou China; ^4^ Department of Pneumology Lanzhou University Second Hospital Lanzhou China

**Keywords:** differentiation, interstitial lung diseases, lung fibroblast, myofibroblast, neutrophil extracellular traps, polymyositis

## Abstract

Excessive neutrophil extracellular trap (NET) formation may contribute to polymyositis (PM)‐associated interstitial lung diseases (ILD), but the underlying mechanism is not fully revealed. In this study, we found that NET accelerated the progression of ILD and promoted pulmonary fibrosis (PF) in vivo. miR‐7 expression was down‐regulated in lung tissue of PM group than control group, and NETs further decreased miR‐7 expression. TLR9 and Smad2 were up‐regulated in lung tissue of PM group than control group, and NETs further increased TLR9 and Smad2 expressions. In vitro experiments showed that PMA‐treated NETs accelerated the proliferation of LF and their differentiation into myofibroblast (MF), whereas DNase I decreased the promotion effect of NETs. Neutrophil extracellular trap components myeloperoxidase (MPO) and histone 3 also promoted the proliferation and differentiation of LF. In addition, we demonstrated that TLR9 involved in the regulation of NETs on LF proliferation and differentiation, and confirmed the interaction between miR‐7 and Smad2 in LF. Finally, miR‐7‐Smad2 pathway was confirmed to be involved in the regulation of TLR9 on LF proliferation and differentiation. Therefore, NETs promote PM‐related ILD, and TLR9‐miR‐7‐Smad2 signalling pathway is involved in the proliferation of LFs and their differentiation into MFs.

## 1 INTRODUCTION

Polymyositis (PM) is the common disease of idiopathic inflammatory myopathy that affects skeletal muscle, lung, heart, joints, etc.^1^ Interstitial lung diseases (ILDs) is the most common complication which seriously affects the prognosis of PM patients.^2^ A retrospective study showed the morbidity rate was 48.9% in patients with PM/dermatomyositis (DM)‐related ILD in Chinese Han population.^3^ Another study reported the mortality rate was 47.1% in myositis patients with ILD.^4^ Based on the high morbidity and mortality rates, finding effective preventive and therapeutic methods is important for improving prognosis of patients with PM‐related ILD. Interstitial lung diseases can ultimately lead to pulmonary fibrosis (PF),^5^ which is characterized by aggregation, proliferation and activation of lung fibroblasts (LF) and excessive deposition of extracellular matrix (ECM) proteins.^6^ Myofibroblast (MF) is a special type of LF, which is characterized by the expression of α‐smooth muscle actin (α‐SMA) and ECM proteins.^7^ LFs can differentiate into MF phenotype, and the activation, proliferation and differentiation of LFs promote the progression of PF.^8^, ^9^ Therefore, we focused on the underlying mechanism of LF to MF differentiation in the progression of PF.

Neutrophil extracellular traps (NETs) are extracellular networks of chromatin coated with histones, myeloperoxidase (MPO) and neutrophil elastase. Neutrophil extracellular traps are released by neutrophils triggered by phorbol myristate acetate (PMA), inflammatory mediators, viruses bacteria, parasites, etc.^10^ Increasing evidence showed that NETs are closely related to autoimmunity disease and pulmonary diseases, such as allergic asthma, cystic fibrosis and acute lung injury.^11^, ^12^ Excessive NET formation is hard to be completely degraded due to the decreased DNase I activity, which may contribute to PM‐associated ILD.^13^, ^14^ NETs can induce the activation of LFs and their differentiation into MFs, and in vitro experiments show that cigarette smoke extract, magnesium silicate or bleomycin stimulate neutrophils to release NETs.^8^ Besides, collagen production, connective tissue growth factor expression and the proliferation are promoted in MFs after the stimulation of NETs.^8^ Therapeutic molecules targeting NETs in pulmonary diseases including DNases, MPO inhibitors, antiproteases and anti‐histone antibodies remarkably decreased fibrosis in NET‐related diseases.^15^ Therefore, NETs play an important role in the activation of LFs and their differentiation into MFs.

Toll‐like receptor 9 (TLR9) is a member of TLR family that recognizes unmethylated cytosine‐guanine dinucleotide (CpG) motifs of endogenous or exogenous DNA, which is exclusively expressed by professional immune cells.^16^ TLR9 expression is up‐regulated in the progression of PF, and TLR9 activation induced differentiation of MFs and exacerbated PF.^17^, ^18^ The concentration of TLR9 is higher in lung tissue from rapidly progressive PF patients than that of slowly progressive PF patients, and LFs from rapid progressive PF patients are more responsive to CpG DNA (TLR9 agonist) than that from slowly progressive PF patients.^19^ Moreover, NETs promoted liver cancer through the activation of TLR9 pathway, and high‐mobility group box 1 (HMGB1) released from NETs facilitated TLR9 activation and the progression of liver cancer.^20^ However, the regulation role of NETs on TLR9 is still not known in PF. Studies have shown that TLR9 exerts its role in liver injury, systemic lupus erythematosus and lung cancer through regulating microRNA expression levels.^21^, ^22^, ^23^ miR‐7 is an immune‐related microRNA that is decreased in serum samples of DM patients.^24^ Besides, TLR9 suppressed miR‐7 expression level to promote PIK3R3/Akt pathway, thus to enhance the growth and metastasis of lung cancer cells.^25^ Therefore, we speculated that NETs‐TLR9‐miR‐7 might involve in the differentiation of MFs in the progression of PF.

Smad2, a downstream molecule of TGF‐β1, belongs to Smad family.^26^ Total and nuclear Smad2 expression is remarkably higher in PF‐derived lung MFs, and Smad2/3 signalling is facilitated in PF‐derived fibroblasts, thus to promote LF to MF differentiation.^27^ miRNAs are found to target Smad2 in the TGF‐β1 signalling cascade and can decrease TGF‐β1–induced MF differentiation.^28^ A recent study showed that TLR9 and p38 signalling played a critical role in the activation of TGF‐β1/Smad2/3/Foxp3 signalling cascade in chronic stress‐induced immune suppression.^26^ Moreover, bioinformatics software predicted there were binding sites between miR‐7 and Smad2 3′UTR. Therefore, we speculated that NETs‐TLR9‐miR‐7‐Smad2 might involve in the differentiation of MFs in the progression of PF.

## 2 MATERIALS AND METHODS

### 2.1 Preparation of rat skeletal muscle homogenate

Sprague‐Dawley (SD) rats were purchased from laboratory animal centre of Lanzhou University. Then, the rats were killed and hindlimb skeletal muscles were collected. Fascia, fat, blood vessel and nervous tissue were removed from the hindlimb skeletal muscles. The tissues were cut into small pieces and immersed in ice‐cold 0.9% sodium chloride injection for 30 minutes. Homogenizing treatment was carried out with T10 high‐speed disperser at a rotating speed of 10 500*g*. Each homogenizing process lasted 15 seconds and remained stationary for 30 seconds. Then, the homogenized liquid was filtered with four layers of gauze, and the filtrate was broken by VCX500 ultrasonic cell breaker. The filtrate was centrifugated by T10 high‐speed disperser at a rotating speed of  1300*g*. The protein level in the supernatant was determined which was 13.1 mg/mL. The supernatant is divided into several standard containers for further use.

### 2.2 Isolation of neutrophils

Mouse neutrophils were isolated from bone marrow and purified by density gradient centrifugation according to the previous report.^29^ Mice were killed, and muscles and joints from the femur and tibia were removed without breaking the bones. Then, epiphyses of the bones were cut, and bone marrow was washed with a 1‐mL syringe at both ends of the bone. Cells were suspended in 1 mL of ice‐cold sterile PBS. Histopaque 1119 (3 mL) was added in a 15‐mL tube at the bottom with 3 mL of Histopaque 1077 overlaid. Then, the bone marrow cell suspension was added on the top of Histopaque 1077 and centrifuged at 700*g* for 30 minutes. Finally, the neutrophils were collected at the interface of the Histopaque 1119 and 1077 layers. The viability and purity of neutrophils were >95% and >90%, respectively.

### 2.3 Animals and induction of PM mice model

Female BALB/c mice (6‐8 weeks, weighed 15‐19 g) were purchased from laboratory animal centre of Lanzhou University and kept in a 12‐hour light/12‐hour dark environment with no limitation to water and food. Mice were randomly divided into control (n = 7), PM (n = 6) and PM+NET groups (n = 5). Mice model of autoimmune inflammatory myopathy was established by using skeletal muscle components as immune inducers. The rat skeletal muscle homogenate (30 mg/mL) was emulsified at a volume ratio of 1:1 with the complete Freund's adjuvant (CFA, 0.25 mL). For PM group, the mixture was subcutaneously injected into both sides of the back of mice with an immunizing dose of 0.5 mL/time (0.25 mL at one side). Immunization was performed on days 0, 7, 14, 21 and 28, respectively. And pertussis toxin (2 μg/0.5 mL) was intraperitoneally injected into the mice at days 0 and 7. The mice were killed at day 35. For control group, a mixture of saline and CFA at a volume ratio of 1:1 was subcutaneously injected into both sides of the back of mice. The rest procedures were the same as PM group. For PM+NET group, the procedures were the same as PM group. Besides, NET was intraperitoneally injected into the mice at days 21 and 28. The mice were killed at day 35. Lung tissues were collected from these groups for pathological section examination. All animal experiments were approved by the Ethics Committee of Lanzhou University Second Hospital.

### 2.4 Pathological examination

After fixation of the lung tissue, the slices were embedded in paraffin. Sections of 3 μm were stained by haematoxylin and eosin (HE) to identify lung structure. For immunohistochemistry, primary antibodies included rabbit anti‐myeloperoxidase antibody (anti‐MPO; Abcam) and anti‐alpha smooth muscle actin antibody (anti‐α‐SMA; Abcam). Horseradish peroxidase‐labelled goat anti‐rabbit IgG (Beyotime Biotechnology) was used as a secondary antibody.

### 2.5 Cell culture, treatment and transfection

Primary LF fibroblast was purchased from the American Type Culture Collection (ATCC). The second‐generation or third‐generation cells were used for the following experiments. Neutrophils were isolated from the whole blood of healthy volunteers. Neutrophil extracellular traps was induced by overnight stimulation of neutrophils with 40 ng/mL of phorbol 12‐myristate 13‐acetate (PMA; Sigma). After centrifugation at 100*g* for 10 minutes, cell debris was removed and supernatants containing NETs were collected. The PMA‐stimulated NETs were used to stimulate LFs in PMA group. Unstimulated NETs were used to cultivate LFs in control group. In PMA+DNase I group, the LFs were cultivated by PMA‐stimulated NETs pre‐treated with DNase I (10 U/mL, Thermo Scientific) in 37°C for 30 minutes. In PMA+MPO inhibitor group, the PMA‐stimulated NETs were pre‐treated with 500 nmol/L MPO inhibitor (Cayman Chemical) for 30 minutes, and then, the NETs were used to cultivate LFs. In PMA+H3 inhibitor group, the PMA‐stimulated NETs were pre‐treated with 1 μg/mL neutralizing peptide for histone 3, and then, the NETs were used to cultivate LFs. In rhMPO group, 10 ng/mL recombinant human MPO (USBiological) was used to stimulate LFs. In rhH3 group, 5 μg/mL recombinant human histone 3 (Sigma) was used to stimulate LFs. In TLR9 agonist CpG‐ODN group, LFs were treated with 1 μmol/L CpG‐ODN (InvivoGen) for 24 hours, and then, PMA‐stimulated NETs were used to stimulate LFs.

Short hairpin RNA for TLR9 (TLR9 shRNA) was designed and synthesized by Ribobio Co., Ltd. miR‐7 mimic, miR‐7 inhibitor and their negative controls (pre‐NC or NC) were purchased from Ribobio Co., Ltd. LFs (2 × 10^5^ cells/well) were cultured in 6‐well plates overnight and transfected with TLR9 shRNA, Ctrl shRNA, miR‐7 mimic, miR‐7 inhibitor or their corresponding negative controls using the Lipofectamine 2000 reagent (Thermo Fisher Scientific).

### 2.6 SYTOX Green nucleic acid stain

PBS‐treated or PMA‐treated neutrophils (1 × 10^9^ cells/L) were suspended in Hanks' Balanced Salt Solution (HBSS; Thermo Fisher Scientific). Then, 250 µL cell suspension was added to 35‐mm glass‐bottom dishes coated with Poly‐l‐Lysine (Sigma), and SYTOX Green Nucleic Acid Stain (Thermo Fisher Scientific) was added at a concentration of 4 nmol/L, and the stained neutrophils were observed by a fluorescence microscope (Olympus).

### 2.7 Quantitative real‐time polymerase chain reaction (qRT‐PCR)

Total RNA was extracted from LFs or lung tissues using TRIzol reagent (Thermo Fisher Scientific) according to the manufacturer's instructions. For quantification of ACTA2, CCN2, ADAM12, miR‐7 and Smad2 expression, total RNA was reverse‐transcripted into cDNA using the High‐Capacity cDNA Reverse Transcription Kit (Thermo Fisher Scientific) according to the manufacturer's instructions. qRT‐PCR was performed with the SuperScript III Platinum SYBR Green One‐Step qRT‐PCR Kit (Thermo Fisher Scientific) according to the manufacturer's instructions. The relative expression of ACTA2, CCN2, ADAM12, miR‐7 and Smad2 was expressed as a function of threshold cycle (Ct) and analysed by 2^−ΔΔCt^ method.

### 2.8 Western blotting

The proteins from lung tissues and LFs were extracted using RIPA buffer containing protease/phosphatase inhibitors (Thermo Fisher Scientific). BCA Protein Assay Kit (Beyotime Biotechnology) was used to quantify the concentration of proteins. After boiling for 5 minutes at 95°C, sample proteins (25 μg) were separated using sodium dodecyl sulphate‐polyacrylamide gel electrophoresis (SDS‐PAGE) and then transferred to polyvinylidene difluoride membranes (PVDF; Merck Millipore). The membranes were blocked with 5% skim milk for 1 hour at room temperature and then incubated with the primary antibodies against TLR9 (1:1000, Abcam), Smad2 (1:500, Abcam), α‐SMA (1:500, Thermo Fisher Scientific), CCN2 (1:1000, Abcam), p‐p38 (1:2000, Thermo Fisher Scientific), p38 (1:1000, Abcam), p‐AKT (1:1000, Thermo Fisher Scientific), AKT (1:500, Abcam), p‐p65 (1:1000, Cell Signaling Technology), p65 (1:500, Abcam), β‐actin (1:500, Abcam) or GAPDH (1:500, Abcam) overnight at 4°C. Then, the membranes were incubated with the horseradish peroxidase‐conjugated secondary antibodies (1:1000, Abcam) for 2 hours at room temperature. Blots were visualized using the ECL Western Blotting Substrate (Thermo Scientific) on ChemiDoc MP Imaging System (Bio‐Rad).

### 2.9 Soluble collagen measurement

Soluble collagens I, II, III and IV in the supernatant of LFs in different groups were detected by Sircol Collagen Assay Kit (Biocolor) according to the manufacturer's instructions after 48 hours of stimulation. The plate was read at 570 nm.

### 2.10 MTT assay

MTT (3‐(4,5‐dimethylthiazol‐2‐yl)‐2,5‐diphenyltetrazolium bromide) assay was used to measure the proliferation of LFs in different groups. LFs (5 × 10^4^) were seeded into a 96‐well plate and cultured overnight. Subsequently, 20 μL MTT (5 mg/mL; Invitrogen) was added to each well and cultured for 4 hours. Then, cells were lysed using dimethylsulphoxide (DMSO, 150 μL/well; Sigma), and the optical density was read at 570 nm.

### 2.11 EdU experiment

BeyoClick EdU‐488 Cell Proliferation Kit was also used to measure the proliferation of LFs in different groups. LFs were seeded in a 24‐well plate at a density of 2 × 10^5^/mL and cultured for 48 hours. Then, cells were stained with 10 μmol/L EdU for an additional 24 hours according to the manufacturer's instruction. Images were obtained using fluorescence microscopy (Leica).

### 2.12 Flow cytometry analysis

Apoptosis analysis was performed with eBioscience Annexin V‐FITC Apoptosis Detection Kit (Thermo Fisher Scientific). The treated LFs were collected and centrifugated at 530*g* for 5 minutes. Cells were resuspended in 1× Annexin V binding buffer (195 μL). Then, the cells were incubated with Annexin V/FITC (5 μL) and PI (10 μL) in the dark for 15 minutes at room temperature. The apoptosis of LFs was analysed using a flow cytometry (FACScan II; BD Biosciences).

### 2.13 Dual‐luciferase reporter gene assay

Dual‐luciferase reporter gene assay was performed to confirm the interaction between miR‐7 and the Smad2 3′UTR. According to the putative miR‐7 binding sites in the Smad2 3′UTR (Smad2‐WT), the Smad2 3′UTR with mutated miR‐7 binding sites (Smad2‐Mut) was generated. The recombinant luciferase reporter vectors containing Smad2‐WT (pGL‐Smad2‐WT) or Smad2‐Mut (pGL‐Smad2‐Mut) were constructed by Ribobio Co., Ltd. Then, pGL‐Smad2‐WT or pGL‐Smad2‐Mut combined with miR‐7 mimic or miR‐7 inhibitor was co‐transfected into LFs using Lipofectamine 2000 reagent (Thermo Fisher Scientific). After 48 hours, luciferase activity was detected using Dual‐Luciferase Assay System (Promega) according to the manufacturer's instructions.

### 2.14 Statistical analysis

Data were from three independent experiments, presented as means ± standard deviations (SD), and analysed by SPSS software (version 19.0, USA). The differences between groups were analysed by *t* test or one‐way analysis of variance (ANOVA) with *P* < .05 considered statistically significant.

## 3 RESULTS

### 3.1 NET facilitated the progression of PM‐related ILD

**Figure 1:**
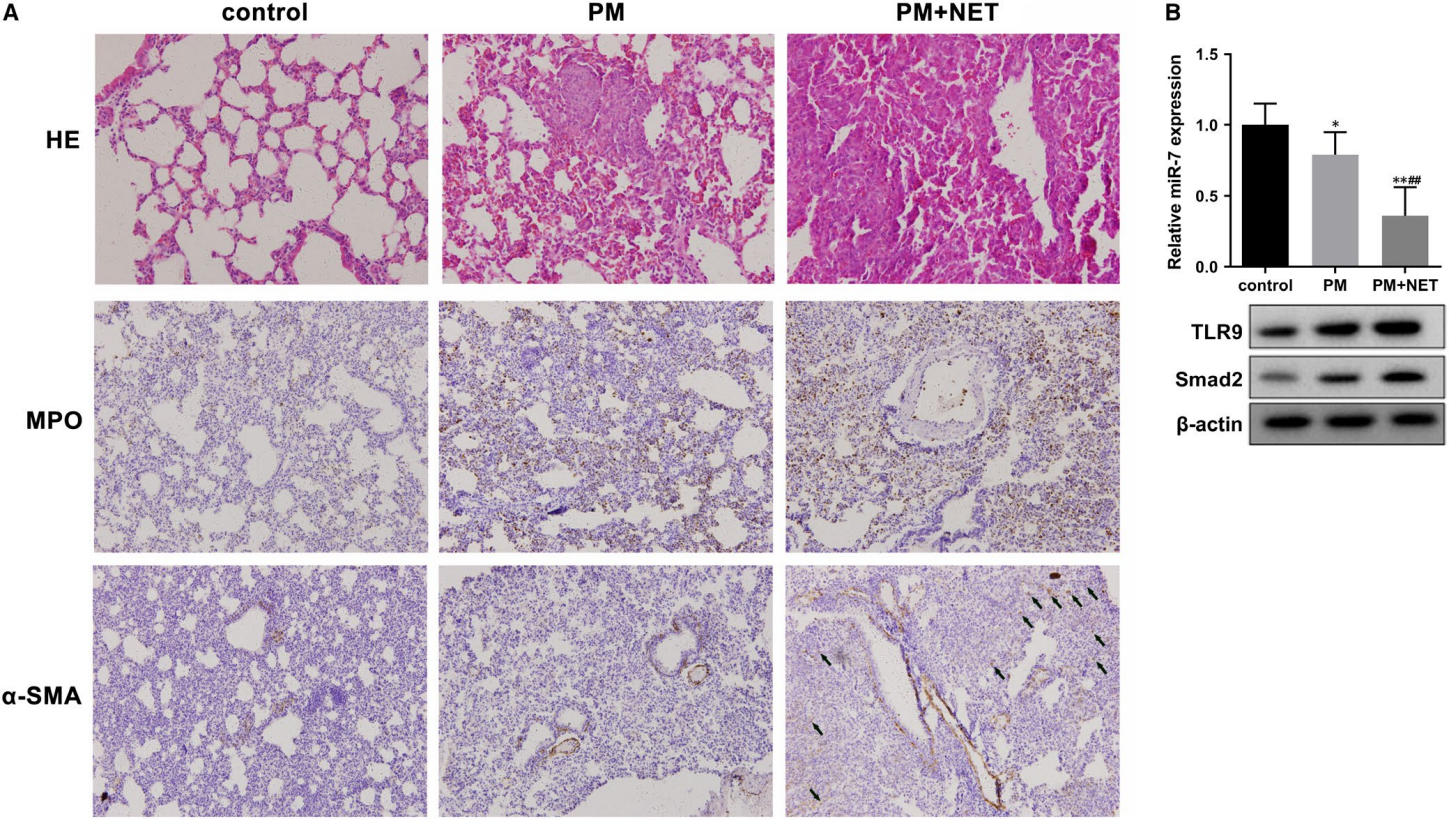
NET facilitated the progression of PM‐ILD. Female BALB/c mice were divided into control group (n = 7), PM group (n = 6) and NET+PM group (n = 5). A, HE staining (×400) of lung tissues in control group, PM group and PM+NET group. Immunohistochemistry (×200) of lung tissues in control group, PM group and PM+NET group. MPO, a marker of NET. α‐SMA, a marker of MF. The black arrow pointed to positive α‐SMA. B, miR‐7, TLR9 and Smad2 expression levels in control, PM and PM+NET groups. *P < .05 vs control, **P < .01 vs control, ## *P* < .01 vs PM

To determine the effect of NET on the progression of PM, PM mice model was established and intraperitoneally injected with NET. HE staining showed normal structure of lung tissue and homogeneous alveolar septum in control group, apparent cell infiltration in pulmonary interstitium and alveolar spaces, oedema and widen of alveolar septum, and obvious pneumonia symptoms in PM group (Figure 1A). In PM+NET group, cell infiltration was lesser than PM group; however, ECM was obvious, and PF symptoms were observed. Immunohistochemistry showed slightly MPO (a marker of NET) expression in control group, and MPO expression in PM+NET group was higher than PM group (Figure 1A). Besides, α‐SMA (a marker of MF) expression in PM+NET group was higher than PM group (Figure 1A). miR‐7 expression was down‐regulated in the lung tissue of PM group and further decreased in PM+NET group (Figure 1B). TLR9 and Smad2 expression levels were up‐regulated in PM group and further increased in PM+NET group (Figure 1B). ILD can result in PF, and the typical pathological process includes early lung epithelial cell injury and obvious inflammatory reaction caused by aggregation of macrophages, neutrophils and lymphocytes in the alveoli. After that, the proliferation of fibroblast was obvious and ECM was deposited, which leads to the formation of honeycomb lung. According to the results of Figure 1, PF occurred in NET+PM group, which indicated that NET could accelerate the progression of ILD and promote PF. Furthermore, in supplementary experimental, miR‐7 mimic inhibited PF in mice from PM/NET+miR‐7 agomir group (Figure S1A) , indicating that miR‐7 overexpression prevents the progression of PM‐related ILD.

### 3.2 NETs accelerated the proliferation of LF and their differentiation into MF

**Figure 2:**
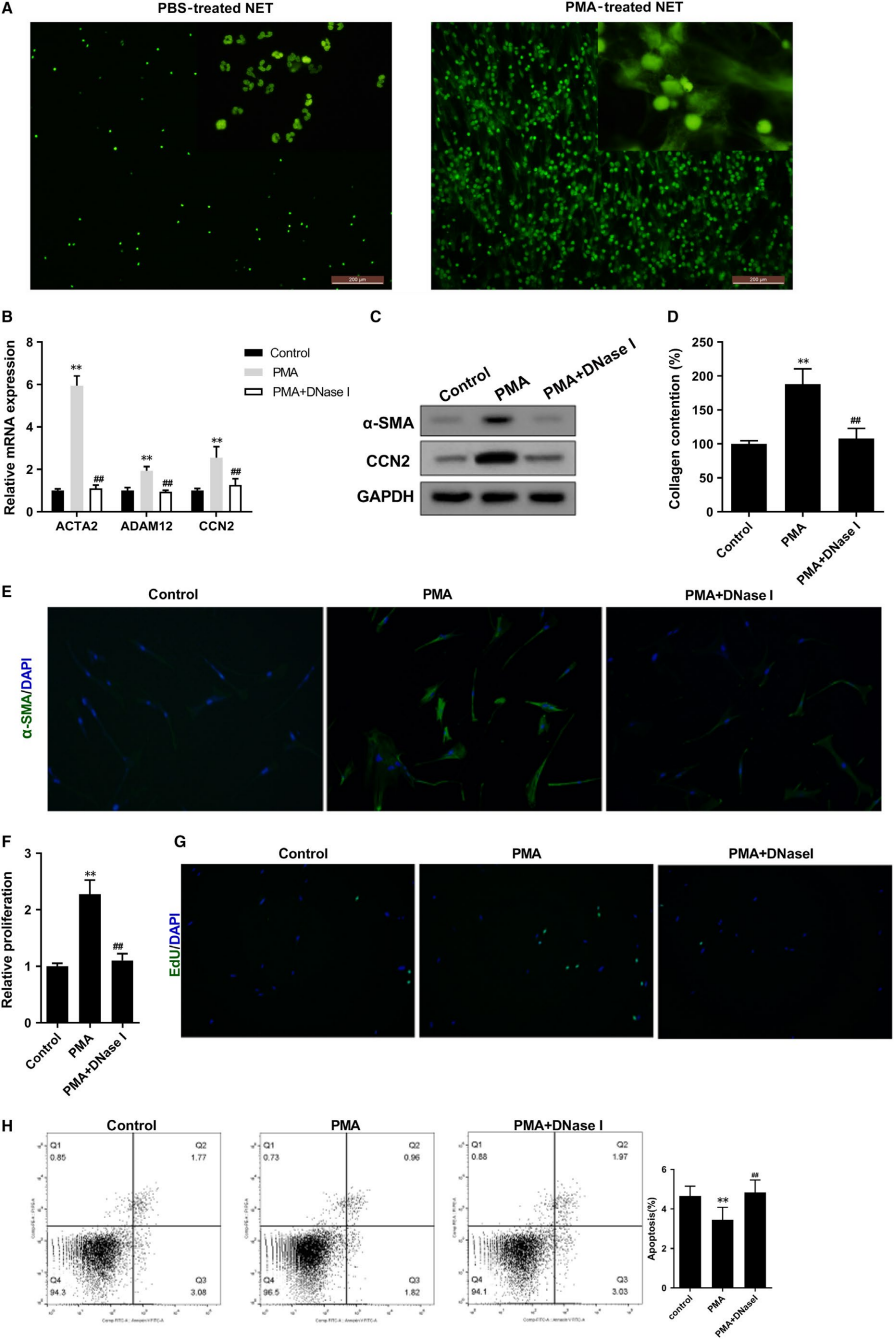
NETs accelerated the proliferation of LF and their differentiation into MF. LFs were divided into control group, PMA group and PMA+DNase I group. A, SYTOX Green nucleic acid stain showed that the nuclear membrane was fractured and DNA was released in PMA‐treated NET. B, qRT‐PCR assay detected mRNA levels of ACTA2 (α‐SMA gene), CCN2 (connective tissue growth factor) and ADAM12 (ADAM metallopeptidase domain 12) in control, PMA and PMA+DNase I groups. C, Western blot assay detected protein levels of α‐SMA and CCN2 in control, PMA and PMA+DNase I groups. D, PMA‐stimulated NET promoted the formation of collagen in LF, whereas the formation of collagen was reduced by DNase I. E‐F, The proliferation of LF was detected by MTT assay. G, The proliferation of LF was also detected by EdU experiment. H, The apoptosis of LF was detected by flow cytometry analysis. **P < .01 vs control, ##*P* < .01 vs PMA

To demonstrate the effect of NET in PF, we explored whether NET could induce the activation of fibroblast in vitro. As shown in Figure 2A, SYTOX Green nucleic acid stain showed that the nuclear membrane was fractured and DNA was released in PMA‐treated NET. We found PMA‐stimulated NET promoted mRNA levels of ACTA2 (α‐SMA gene), CCN2 (connective tissue growth factor gene, a profibrotic mediator) and ADAM12 (ADAM metallopeptidase domain 12, a fibrotic component) in LFs, whereas DNase I decreased these expressions, which indicated DNase I relieved PF (Figure 2B). Protein levels of α‐SMA and CCN2 were up‐regulated in PMA group, whereas DNase I decreased the protein levels of α‐SMA and CCN2 (Figure 2C). PMA‐stimulated NET promoted the formation of collagen in LF, whereas the formation of collagen was reduced by DNase I (Figure 2D). Moreover, PMA‐stimulated NET promoted the proliferation of LF (Figure 2E‐G) and inhibited the apoptosis of LF (Figure 2H), whereas DNase I suppressed the proliferation of LF and promoted LF apoptosis (Figure 2E‐H).

### 3.3 Effects of NET components MPO and histone 3 on the proliferation and differentiation of LF

**Figure 3:**
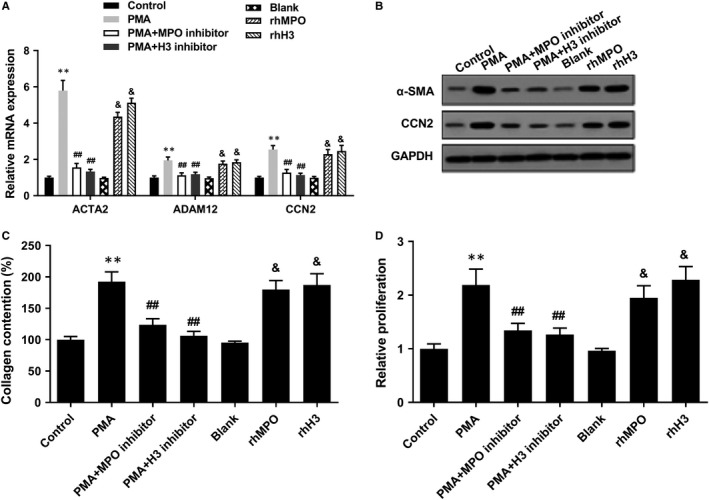
Effects of NET components MPO and histone 3 on the proliferation and differentiation of LF. LFs were divided into control, PMA, PMA+MPO inhibitor, PMA+H3 inhibitor, blank, rhMPO and rhH3 groups. A, Compared with PMA group, MPO inhibitor or H3 inhibitor‐treated NET decreased mRNA levels of ACTA2, CCN2 and ADAM12 in LFs. B, Compared with PMA group, MPO inhibitor or H3 inhibitor‐treated NET down‐regulated protein levels of α‐SMA and CCN2 in LFs. C, Compared with PMA group, MPO inhibitor or H3 inhibitor‐treated NET reduced collagen production of LFs. D, Compared with PMA group, MPO inhibitor or H3 inhibitor‐treated NET suppressed the proliferation of LFs, whereas rhMPO and rhH3 promoted mRNA levels of ACTA2, CCN2 and ADAM12, protein levels of α‐SMA and CCN2, collagen production and proliferation of LFs. **P < .01 vs control, ##*P* < .01 vs PMA, &&*P* < .01 vs blank

We further detected the effects of the components of NET on the proliferation and differentiation of LF. Compared with PMA group, MPO inhibitor or H3 inhibitor‐treated NET decreased mRNA levels of ACTA2, CCN2 and ADAM12 in LFs (Figure 3A), down‐regulated protein levels of α‐SMA and CCN2 in LFs (Figure 3B), reduced collagen production of LFs (Figure 3C) and suppressed the proliferation of LFs (Figure 3D). Whereas rhMPO and rhH3 promoted mRNA levels of ACTA2, CCN2 and ADAM12, protein levels of α‐SMA and CCN2, collagen production and proliferation of LFs (Figure 3A‐D). These findings indicated that the components of NET (MPO and histone 3) promoted the proliferation and differentiation of LF.

### 3.4 TLR9 signalling pathway, and miR‐7 and Smad2 expressions in NET‐treated LFs

**Figure 4:**
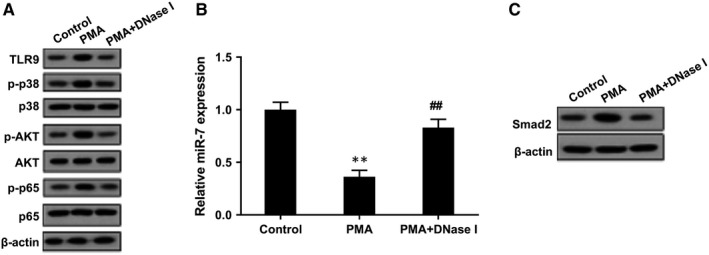
TLR9 signalling pathway, miR‐7 and Smad2 expressions in NET‐treated LFs. LFs were divided into control group, PMA group and PMA+DNase I group. A, Western blot assay showed that TLR9 protein level and phosphorylation levels of its downstream molecules p38, AKT and p65 were up‐regulated in PMA group, whereas PMA+DNase I decreased these expressions. B, miR‐7 expression level was down‐regulated in PMA group, whereas PMA+DNase I increased miR‐7 expression. C, Smad2 protein level was up‐regulated in PMA group, whereas PMA+DNase I inhibited Smad2 expression. **P < .01 vs control, ##*P* < .01 vs PMA

Next, we explored the mechanism of NETs in the regulation of LFs. As shown in Figure 4A, TLR9 protein level and phosphorylation levels of its downstream molecules p38, AKT and p65 were up‐regulated in PMA group, whereas PMA+DNase I decreased these expressions. miR‐7 expression level was down‐regulated in PMA group, whereas PMA+DNase I increased miR‐7 expression (Figure 4B). Smad2 protein level was up‐regulated in PMA group, whereas PMA+DNase I inhibited Smad2 expression (Figure 4C).

### 3.5 TLR9 involved in the regulation of NETs on LF proliferation and differentiation

**Figure 5:**
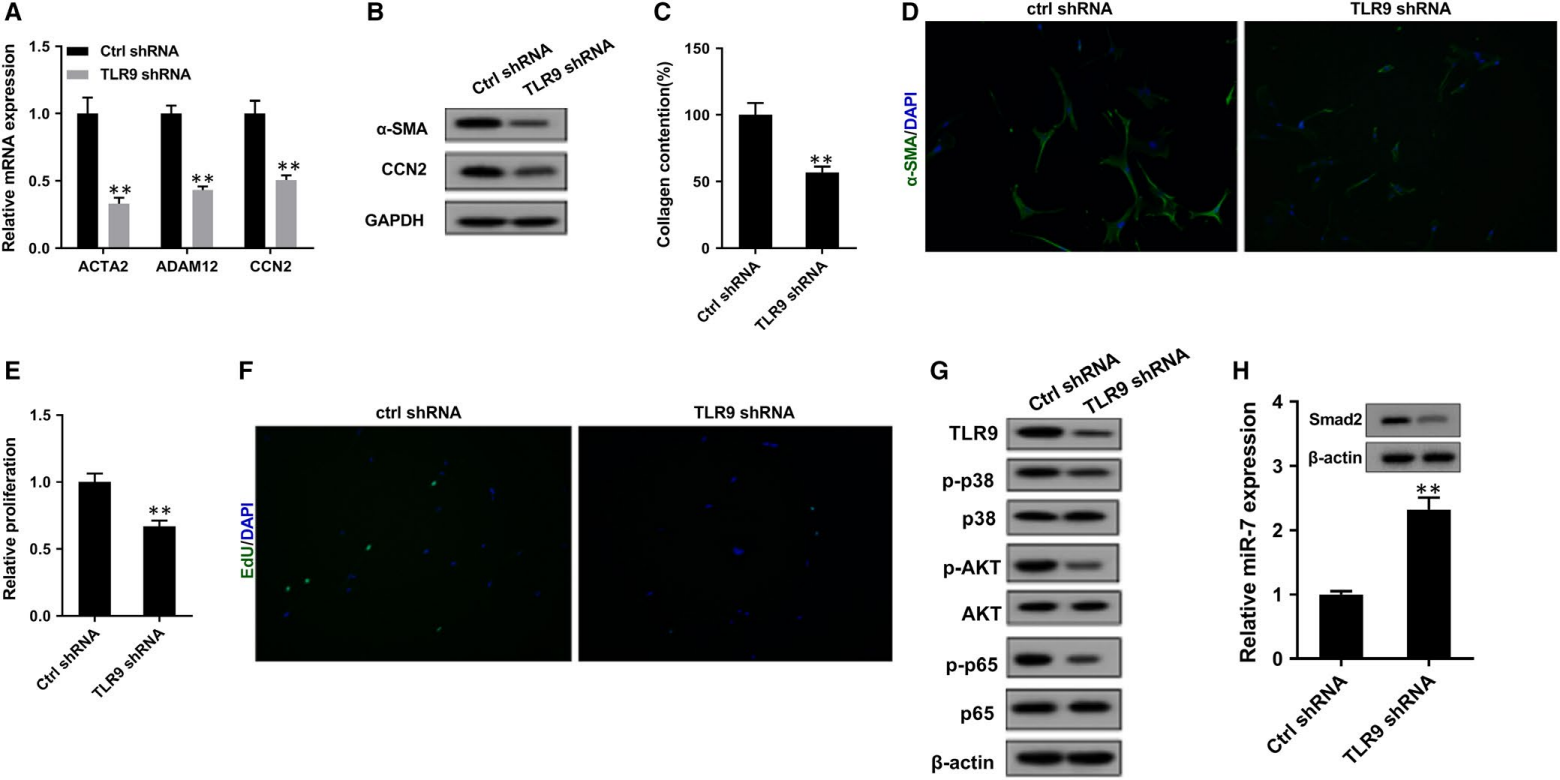
TLR9 involved in the regulation of NETs on LF proliferation and differentiation. LFs were transfected with Ctrl shRNA and TLR9 shRNA. Then, PMA‐stimulated NETs were used to cultivate LFs. A, Compared with Ctrl shRNA group, mRNA levels of ACTA2, CCN2 and ADAM12 were down‐regulated in TLR9 shRNA group. B, Compared with Ctrl shRNA group, protein levels of α‐SMA and CCN2 were down‐regulated in TLR9 shRNA group. C, Compared with Ctrl shRNA group, collagen production was inhibited in TLR9 shRNA group. D‐E, The proliferation of LFs was detected using MTT assay. F, The proliferation of LF was also detected by EdU experiment. G, Compared with Ctrl shRNA group, TLR9 protein level and phosphorylation levels of its downstream molecules p38, AKT and p65 were inhibited in TLR9 shRNA group. H, Compared with Ctrl shRNA group, miR‐7 expression level was up‐regulated in TLR9 shRNA group, and Smad2 protein level was down‐regulated. **P < .01 vs Ctrl shRNA

To determine the role of TLR9 in the regulation of NETs on LF, LFs were transfected with Ctrl shRNA and TLR9 shRNA. We found TLR9 shRNA decreased mRNA levels of ACTA2, CCN2 and ADAM12 in LFs (Figure 5A), down‐regulated protein levels of α‐SMA and CCN2 in LFs (Figure 5B), inhibited collagen production of LFs (Figure 5C), suppressed the proliferation of LFs (Figure 5D‐F), decreased TLR9 protein level and phosphorylation levels of its downstream molecules p38, AKT and p65 (Figure 5G), up‐regulated miR‐7 expression level and down‐regulated Smad2 protein level in LFs (Figure 5H).

### 3.6 The regulation role of miR‐7 on Smad2 in LF

**Figure 6:**
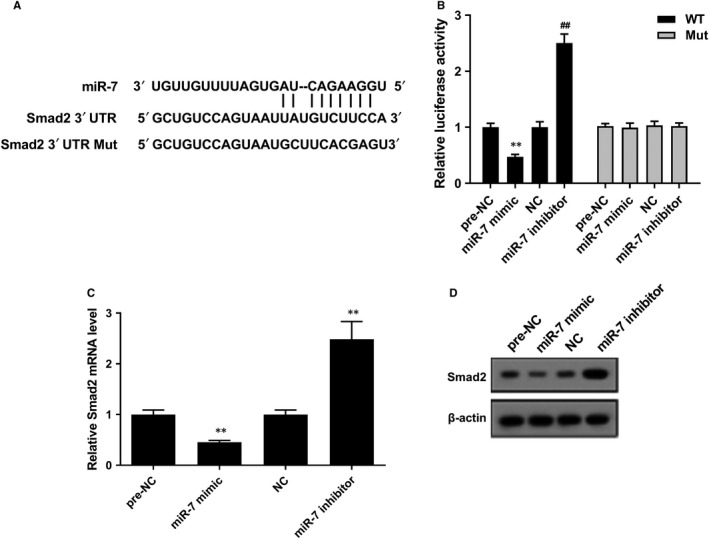
The regulation role of miR‐7 on Smad2 in LF. A, Bioinformatics software predicted the binding sites between miR‐7 and Smad2 3′UTR. B, Dual‐luciferase reporter gene assay showed that miR‐7 mimic reduced the luciferase activity of Smad2 3′UTR WT, and miR‐7 inhibitor increased the luciferase activity of Smad2 3′UTR WT. C, miR‐7 mimic decreased mRNA level of Smad2, and miR‐7 inhibitor up‐regulated mRNA level of Smad2. D, miR‐7 mimic down‐regulated protein level of Smad2, and miR‐7 inhibitor up‐regulated protein level of Smad2. **P < .01 vs pre‐NC or NC

As shown in Figure 6A, bioinformatics software predicted the binding sites between miR‐7 and Smad2 3′UTR. Besides, miR‐7 mimic significantly reduced the luciferase activity of Smad2 3′UTR WT, and miR‐7 inhibitor significantly increased the luciferase activity of Smad2 3′UTR WT (Figure 6B). There was no significant difference in the luciferase activity of Smad2 3′UTR Mut. Moreover, miR‐7 mimic decreased mRNA level and protein level of Smad2 in LFs, and miR‐7 inhibitor up‐regulated mRNA level and protein level of Smad2 in LFs (Figure 6C,D). These finding confirmed the interaction between miR‐7 and Smad2, and proved Smad2 expression was negatively regulated by miR‐7.

### 3.7 miR‐7‐Smad2 involved in the regulation of TLR9 on LF proliferation and differentiation

**Figure 7:**
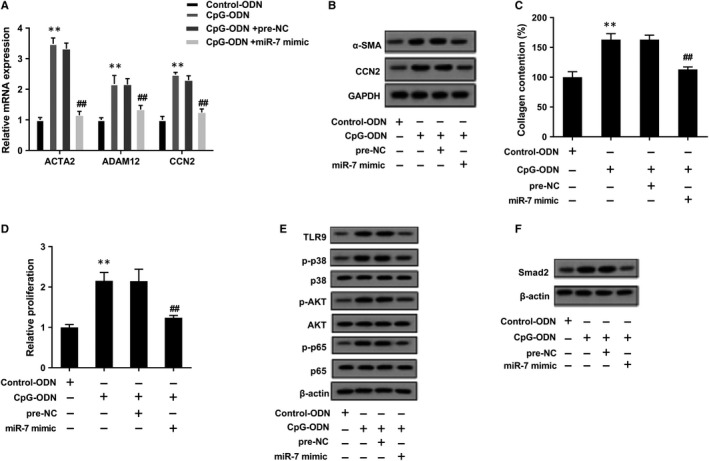
miR‐7‐Smad2 involved in the regulation of TLR9 on LF proliferation and differentiation. LFs were divided into Control‐ODN group, CpG‐ODN (TLR9 agonist) group, CpG‐ODN +pre‐NC group and CpG‐ODN +miR‐7 mimic group. Then, PMA‐stimulated NETs were used to stimulate LFs. A, CpG‐ODN increased mRNA levels of ACTA2, CCN2 and ADAM12, and miR‐7 mimic reversed CpG‐ODN–induced promotion effects. B, CpG‐ODN up‐regulated protein levels of α‐SMA and CCN2, and miR‐7 mimic reversed CpG‐ODN–induced promotion effects. C, CpG‐ODN promoted collagen production of LFs, and miR‐7 mimic reversed CpG‐ODN–induced promotion effect. D, CpG‐ODN accelerated the proliferation of LFs, and miR‐7 mimic reversed CpG‐ODN–induced promotion effect. E, CpG‐ODN increased TLR9 protein level and phosphorylation levels of its downstream molecules p38, AKT and p65, and miR‐7 mimic reversed CpG‐ODN–induced promotion effect. F, CpG‐ODN up‐regulated protein level of Smad2, and miR‐7 mimic reversed CpG‐ODN–induced promotion effect. **P < .01 vs Control‐ODN, ##*P* < .01 vs CpG‐ODN +pre‐NC

As shown in Figure 7A, TLR9 agonist CpG‐ODN increased mRNA levels of ACTA2, CCN2 and ADAM12, and miR‐7 mimic reversed CpG‐ODN–induced promotion effects. CpG‐ODN up‐regulated protein levels of α‐SMA and CCN2, and miR‐7 mimic reversed CpG‐ODN–induced promotion effects (Figure 7B). In addition, CpG‐ODN promoted collagen production of LFs and accelerated the proliferation of LFs, and miR‐7 mimic reversed CpG‐ODN–induced promotion effects (Figure 7C,D). CpG‐ODN could increase TLR9 protein level and phosphorylation levels of its downstream molecules p38, AKT and p65, and miR‐7 mimic reversed CpG‐ODN–induced promotion effect (Figure 7E). We also determined CpG‐ODN up‐regulated protein level of Smad2, and miR‐7 mimic reversed CpG‐ODN–induced promotion effect (Figure 7F). These findings indicated that miR‐7‐Smad2 pathway was involved in the regulation of TLR9 on LF proliferation and differentiation. As shown in Figure S1B, miR‐7 expressions in PM and PM+NET groups were significantly down‐regulated than control group. miR‐7 expression in PM/NET+miR‐7 agomir group was significantly higher than PM/NET+agomir NC group. TLR9 expressions were slightly up‐regulated in PM+NET group and PM/NET+agomir NC group than control group. Smad2 expression was only down‐regulated in PM/NET+miR‐7 agomir group than PM/NET+agomir NC group. Therefore, only miR‐7 could be used as a marker molecule for the progression of PF complicated by PM.

## 4 DISCUSSION

In this study, we provide evidence for the effect of NETs on PM‐related ILD in vivo. Our experimental data suggested that NETs promoted the activation of LFs and their differentiation into MFs in vitro. We also determined the underlying mechanism of NETs on LFs differentiate into MFs in vitro, and demonstrated that TLR9‐miR‐7‐Smad2 signalling pathway involved in the activation of LFs and their differentiation into MFs.

Increasing evidence has shown that NETs and their components cause damage of endothelial and epithelial cells, thus to impair pulmonary function and accelerate the progress of chronic obstructive pulmonary disease.^30^, ^31^, ^32^ NETs also involve in the development of inflammation through inducing the release of cytokine by airway epithelial cells.^33^ Excessive neutrophils and NETs increase the permeability of alveolar epithelial cells and promote lung injury in severe influenza A infection.^34^ Recently, emerging evidence showed that NETs contributed to PM‐associated ILD.^8^, ^13^ NETs might be pathogenesis of PM, which initiated and aggravated ILD.^14^ Moreover, NETs induced the activation of LFs and their differentiation into MFs in vitro*.* 8 These data are in agreement with our results indicating that NETs facilitated the progression of PM‐related ILD and accelerated the proliferation of LFs and their differentiation into MFs.

Even though the promotion role of NETs in the proliferation of LFs and their differentiation into MFs has been confirmed, the underlying mechanism is still not known. It has been reported that histones and HMGB1 induced NET formation through TLR9 and TLR4 signalling pathway; therefore, NET formation is dependent on TLR signalling in liver ischaemia/reperfusion (I/R) injury.^35^ Wan et al^36^ demonstrated that TLR2 and TLR4 agonists enhanced NET formation induced by *S aureus*, and dexamethasone modulated *S aureus*‐induced NET formation through TLR2 and TLR4. Khafaji et al^37^ found that superoxide induced NETs in a TLR4 and neutrophil NADPH oxidase‐dependent pathway after liver I/R. Importantly, Samer et al^20^ discovered that in liver metastases, NETs promoted tumour growth through activation of TLR9 pathway, and MC38 cells deficient in TLR9 remarkably decreased tumour growth in colorectal liver metastasis mice. Therefore, TLR signalling pathway is important in the regulation of NET formation, and NETs may also play a positive role in TLR signalling pathway.

Researchers also discovered that TLR signalling pathway played critical roles in LF to MF differentiation. For example, lipopolysaccharide decreased α‐SMA expression through activating TLR4, thus to prevent cardiac fibroblasts to cardiac MF differentiation.^38^ Mogroside IIIE protected against bleomycin‐induced PF in vivo, and in vitro experiments further determined that MGIIIE suppressed fibroblast activation and collagen production through down‐regulating TLR4/MyD88 MAPK signalling pathway.^39^ TLR9 stimulation facilitated the proliferation and differentiation of cardiac fibroblasts through activating NF‐κB pathway to protect hearts from cardiac rupture.^40^ In this study, we determined the NETs promoted TLR9 signalling pathway in LFs, and TLR9 knockdown suppressed the proliferation of LF and their differentiation into MF. In terms of novel aspects of the present study, to the best of our knowledge, no other studies have demonstrated the role of NETs‐TLR9 in the modulation of the proliferation of LF and their differentiation into MF. These findings enrich the literatures and provide directions for the treatment of PF.

Our data clearly determined that TLR9 agonist CpG‐ODN remarkably up‐regulated CCN2 and α‐SMA expression levels, accelerated the collagen production and promoted the proliferation of LFs, resulting in the promotion of MF differentiation. However, miR‐7 overexpression reversed the promotion effect of CpG‐ODN. Studies have shown that miRNAs are abnormally expressed in PF and exert promotion or suppression functions in PF, such as miR‐9, miR‐21 and miR‐29.^41^, ^42^ miR‐7 is down‐regulated in serum samples of DM patients than that of normal individuals.^24^ In lung cancer, TLR9 suppressed miR‐7 expression level to enhance the growth and metastasis of tumour.^25^ These researches indicated that miR‐7 might involve in the progression of PF. In the present study, the expression levels of miR‐7 and Smad2 were first determined in LFs, and the role of miR‐7‐Smad2 in the regulation of LF proliferation and their differentiation into MF was first identified by in vitro experiments. These findings enrich the literatures and illustrated the possible mechanism of TLR9‐miR‐7‐Smad2 in NET‐mediated LF proliferation and their differentiation into MF.

In conclusion, our data showed that NETs facilitate the progression of PM‐related ILD. In addition, NETs and their components play a promotion role in the activation of LFs and their differentiation into MFs, and TLR9‐miR‐7‐Smad2 signalling pathway is involved in NET‐mediated LF proliferation and their differentiation into MFs. Therefore, our findings can represent potential therapeutic targets for PF.

## CONFLICT OF INTEREST

None.

## AUTHOR CONTRIBUTION

Z SG and S HL participated in the experimental design, manuscript writing and manuscript revision. Z SG, J XQ, Z QY, Z L, Y J and H CH participated in animal experiment, histological experiment, acquisition of data, data analysis and interpretation. S JN, J X and L JY modified grammar and polished the manuscript. All authors read and approved the final manuscript.

## Data Availability

The data sets used and/or analysed during the current study are available from the corresponding author on reasonable request.
