# Supplementary figures and images for: Neutrophil extracellular traps activate lung fibroblast to induce polymyositis‐related interstitial lung diseases via TLR9‐miR‐7‐Smad2 pathway
Source: J Cell Mol Med. 2019 Dec 10;24(2):1658–69. doi: 10.1111/jcmm.14858 (PMC6991674; doi:10.1111/jcmm.14858)

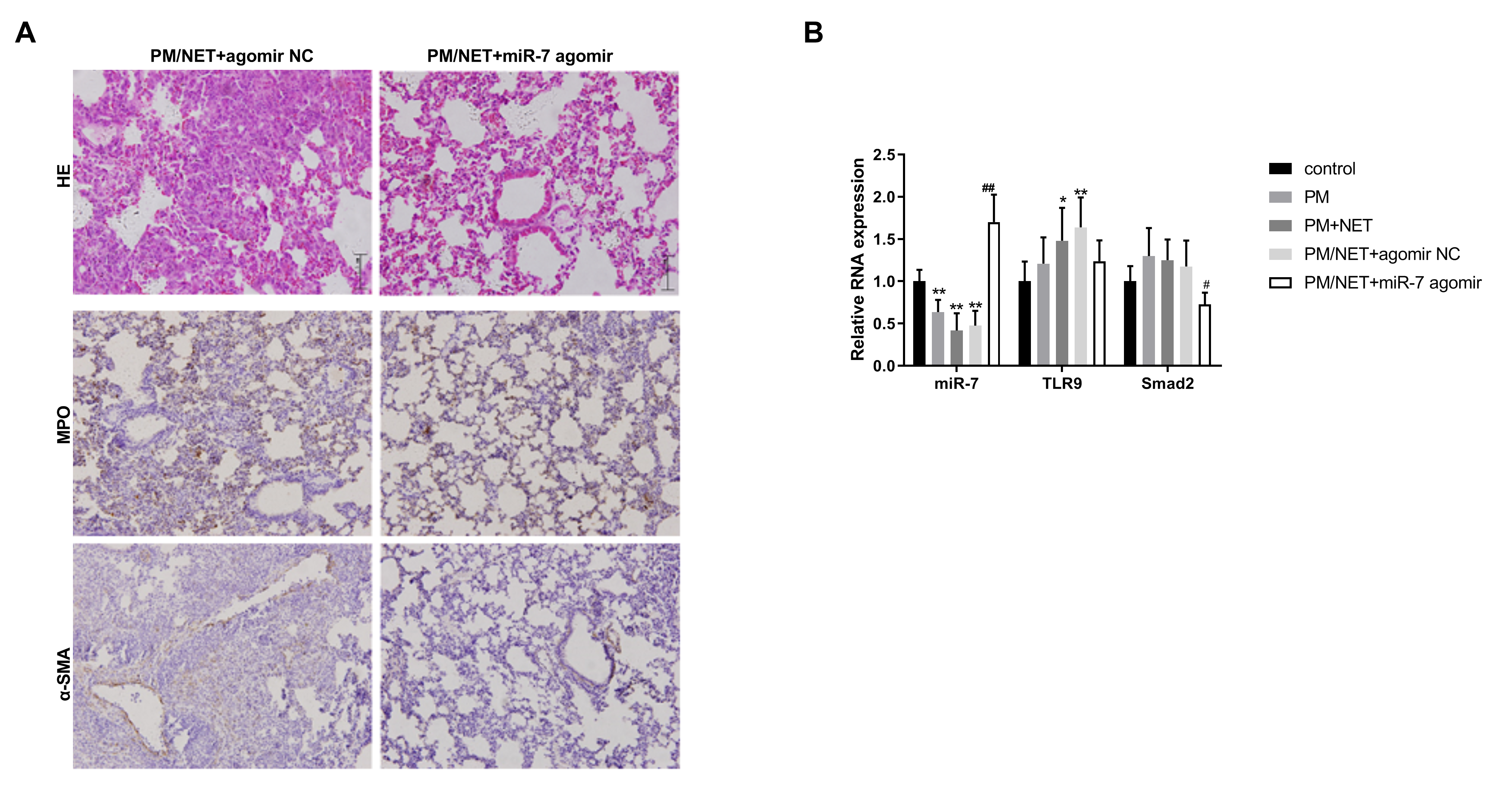

Supplement: Supplementary file 1 [file JCMM-24-1658-s001.tif]
