# Supplementary material for: Neutrophil extracellular traps activate lung fibroblast to induce polymyositis‐related interstitial lung diseases via TLR9‐miR‐7‐Smad2 pathway
Source: J Cell Mol Med. 2019 Dec 10;24(2):1658–69. doi: 10.1111/jcmm.14858 (PMC6991674; doi:10.1111/jcmm.14858)
Supplement: Supplementary file 2 [file JCMM-24-1658-s002.docx]

Supplementary Figure 1. miR-7 overexpression relieved pulmonary fibrosis. For PM/NET+agomir NC group and PM/NET+miR-7 agomir group, miR-7 agomir (150 nmol/kg) or agomir NC (150 nmol/kg) was injected into the mice by caudal vein at day 7. The rest procedures were the same as PM+NET group. The mice were killed at day 35. A. MPO and α-SMA expressions were detected in the lung tissue of mice from PM/NET+agomir NC group (n=5) and PM/NET+miR-7 agomir group (n=5). B. Serum expression levels of TLR9, miR-7 and Smad2 were detected in control, PM, PM+NET, PM/NET+agomir NC, and PM/NET+miR-7 agomir groups. *p<0.05, **p<0.01 vs control; #p<0.05, ##p<0.01 vs PM/NET+agomir NC.
